# Inducible nitric oxide synthase as a potential blood-based biomarker in inflammatory bowel diseases 

**Published:** 2018

**Authors:** Saeed Baranipour, Azade Amini Kadijani, Durdi Qujeq, Shabnam Shahrokh, Mehrdad Haghazali, Alireza Mirzaei, Hamid Asadzadeh-Aghdaei

**Affiliations:** 1 *Student Research Committee, Babol University of Medical Sciences, Babol, Iran*; 2 *Basic and Molecular Epidemiology of Gastrointestinal Disorders Research Center, Research Institute for Gastroenterology and Liver Diseases, Shahid Beheshti University of Medical Sciences, Tehran, Iran*; 3 *Cellular and Molecular Biology Research Center, Health Research Institute, Babol University of Medical Sciences, Babol, Iran*; 4 *Gastroenterology and Liver Diseases Research Center, Research Institute for Gastroenterology and Liver Diseases, Shahid Beheshti University of Medical Sciences, Tehran, Iran*; 5 *Rajaie Cardiovascular Medical and Research Center, Iran University of Medical Sciences, Tehran, Iran*; 6 *Bone and Joint Reconstruction Research Center, Shafa Orthopedic Hospital, Iran University of Medical Sciences, Tehran, Iran*

**Keywords:** Inflammatory bowel diseases, Inducible nitric oxide synthase, Biomarker

## Abstract

**Aim:**

Here, we evaluated the role of (iNOS) as a blood-based biomarker of inflammatory bowel diseases (IBD).

**Background:**

Up-regulation of inducible nitric oxide synthase (iNOS) in the intestinal epithelial cells has been closely associated with the initiation and maintenance of intestinal inflammation in IBD.

**Methods:**

In a case-control design, 59 IBD patients and 30 healthy control subjects were participated in this study. A total of 10 ml blood sample was taken from each participant. Blood leukocytes were isolated and iNOS mRNA expression level was evaluated in the isolated leukocytes using relative quantitative Real-time PCR.

**Results:**

The patients’ population included 40 ulcerative colitis (UC) and 19 Crohn's disease (CD) patients. The flare and remission phase of disease were seen in 43 and 16 patients, respectively. The mean iNOS mRNA expression was not significantly different between the IBD patients and healthy controls (p=0.056). The mean iNOS mRNA expression was significantly higher in the flare phase of the disease compared to the remission phase (p=0.039). No significant difference was observed between the mean iNOS mRNA expression in the blood leukocytes of UC and CD patients (p=0.82).

**Conclusion:**

iNOS is differently expressed in the blood leukocytes of active vs. inactive IBD disease. Thus, it could be potentially used as a non-invasive blood-based biomarker of IBD.

## Introduction

 Inflammatory bowel diseases (IBD), including Crohn disease (CD) and ulcerative colitis (UC), are defined as the chronic intestinal inflammation caused by the host-microbial interactions in a genetically susceptible individual (1). Increased prevalence of IBD in developing countries suggests an epidemiologic evolution for IBD in association with urbanization and industrialization (2). According to the Hong Kong’s registry system, a 6-fold increase in the incidence of CD has been recorded from 1985 to 2014 (2). Considering the growing trend in the incidence of IBD, increasing efforts are being made to identify new biomarkers capable of discriminating different types of IBD, predicting responses to treatment, and help in the differential diagnosis, treatment planning and prognosis prediction (3).

Inducible nitric oxide synthase (iNOS), encoded by the NOS2 gene, triggers the nitric oxide (NO) production in response to pro-inflammatory cytokines. Subsequently, NO contributes to the antimicrobial and antipathogenic activities (4). Yet, the production of NO via the up-regulation of iNOS contains variety of other effects, which may be either detrimental or beneficial depending on the amount, duration and anatomical site of production. In some conditions such as in IBD, iNOS-mediated NO production may become part of a dysregulated immune response, leading to the chronic inflammatory disorders. Increased production of NO in the intestinal epithelial cells following the up-regulation of iNOS has been closely associated with the initiation and maintenance of intestinal inflammation in IBD (5).

Preliminary studies using flow cytometry and western blotting have revealed an increased iNOS level in the circulating monocytes of IBD patients that was also associated with disease activity (6). We hypothesized that the evaluation of iNOS expression using a more convenient laboratory method such as the polymerase chain reaction (PCR) may serve its clinical implication more effectively. Here, we aimed to study the mRNA expression of iNOS in the circulating leukocytes of IBD patients in comparison with healthy control, and to find how this expression associates with the disease characteristics in IBD. 

## Methods

This study was approved by the institutional review board of Shahid Beheshti University of Medical Sciences under the code of IR.SBMU.RIGLD.REC.1396.166. Written informed consent was obtained from the patients before their inclusion in the study. In a case-control design, IBD patients who their diagnosis was confirmed by colonoscopy were entered into the study. The disease phase (flare or remission) was identified through a combination of clinical and endoscopic data (7, 8). All evaluations were performed by one senior specialist. Patients with systemic or local infection, other chronic inflammatory disorders such as osteoarthritis, autoimmune disorders such as rheumatoid arthritis, and concomitant disorders affecting the iNOS expression such as cancer, cardiovascular disorders, diabetes, hypertension, etc, were excluded from the study. Finally, from a total of 68 IBD patients, 59 patients were identified as eligible for the study. A number of 30 healthy subjects were also recruited as the control group of the study.

A total of 10 ml blood sample was taken from each patient and control subject. After the collection of blood samples, ammonium chloride solution was used for the lysis of red blood cells. Subsequently, the blood leukocytes were isolated using the gradient centrifugation. The isolated ‎leukocytes were stored at -80 °C for later examinations.‎

**Relative quantitative Real-time PCR.**

iNOS mRNA expression were evaluated by relative quantitative ‎Real-time PCR method. Following the RNA extraction from the isolated blood leukocytes (YTA RNA Extraction kit, Yekta Tajhiz Azma, Tehran, Iran), the extracted RNA was used as the template for the reverse transcription into complementary DNA (Revert Aid First Strand cDNA Synthesis Kit, Thermo Scientific, USA). Subsequently, the Real-time PCR was done using SYBR® Premix Ex Taq™ II (Takara, Japan) on a Real-Time PCR System Qiagen Rotor-Gene Q. The Real-time PCR protocol was 1 cycle at 95 °C for 2 min (initial denaturation) followed by 40 cycles at 95 °C for 5s (denaturation) and 60 °C for 30s (annealing/extension). Melt curve analysis was used to confirm the specificity of reactions. At the end, the obtained data were processed with the comparative Ct method based on the previously standardized protocol. Accordingly, *2*^−ΔCt ^was computed for each patient and control sample, in which the ΔCt was considered as the CT gene of interest−CT internal control (9).

β-2 microglobulin (B2M) was used as the internal reference gene. The sequences of primers for B2M were as follows: B2M F: TGC TGT CTC CAT GTT TGA TGT ATCT, B2M R: TCT CTG CTC CCC ACC TCT AAGT (10). iNOS primers were designed by one of the authors (AAK). The sequences of primers were as follows: iNOS F: ACA CAG CGT ACC TGA ATT, iNOS R: CCT GGC AAT GGA GAG AAA.

**Statistical analysis**

IBM SPSS for Windows, version 16 were used for the statistical analysis of the data. Descriptive statistics were demonstrated as the mean and standard deviation (SD) or number and percentage (%). Normal distribution of the variables was tested with Kolmogorov–Smirnov test. Independent *t*-test or its nonparametric counterpart (Mann-Whitney U test) was used for the comparison of mean in parametric or nonparametric variables, respectively. Potential correlations were assessed with Pearson’s or Spearman’s correlation coefficient test. A p-value of < 0.05 was considered a significant difference. 

## Results

A total of 59 IBD patients including 19 (32.2%) CD and 40 (67.8%) UC patients with the mean age of 35.76±12.5 years were included in the study. The patients’ population included 38 (64.4%) males and 21 (35.6%) females. The flare phase of the disease was identified in 43 (72.9%) patients. The mean body mass index (BMI) of the patients was 22.6±6.2 kg/m^2^‎. The mean disease duration was 52.9±56.1 months (range 6-216 months). A total of 30 healthy control subjects including 21 (70%) males and 9 (30%) females with the mean age of 32.9±9.6 years were also included. No significant difference was observed between the demographic characteristics of the patients and controls including age (p=0.0.55), BMI (p=0.41) and gender (p=0.34). The clinicodemographic characteristics of the patients are demonstrated in detail in Table 1. 

**Figure 1:**
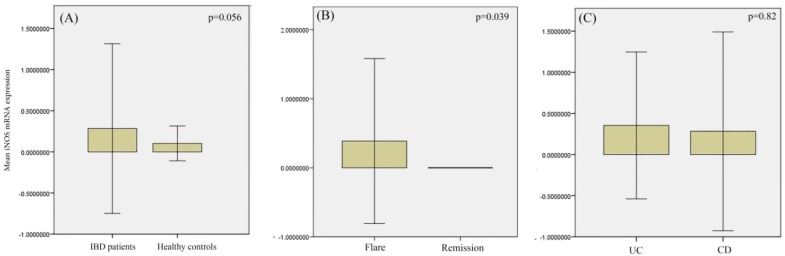
Comparison of the mean iNOS mRNA expression between: (A) the IBD patients and healthy controls; (B) the flare and remission phase of IBD; and (C) UC and CD patients. P-value <0.05 is considered significant

The mean iNOS mRNA expression was 0.3±1 in IBD patients and 0.1±0.2 in the control group (p=0.056) (Figure 1A). The mean iNOS mRNA expression was 0.39±1.2 in the flare and 0.001±0.002 in the remission phase of the disease (p=0.039) (Figure 1B). The mean iNOS mRNA expression was 0.35±0.9 in CD patients and 0.28±1.2 in UC patients (p=0.82) (Figure 1C). No significant association was found between the iNOS mRNA expression and the sex of participants (p=0.16). No significant correlation was found between the iNOS mRNA expression and the disease duration of the patients as well (r=-0.179, p=0.27). Moreover, no significant correlation was found between the iNOS mRNA expression and the demographic characteristics of the patients including the age (r=0.128, p=0.26) and BMI (r=0.112, p=0.38).

**Table 1:** The clinicodemographic characteristics of IBD patients

Variable	IBD Patients (n=59)
Age (year)	‎‎35.76±12.5 ‎
Sex Male Female	‎‎38 (64.4%) ‎ ‎21 (35.6%) ‎
BMI (kg/m^2^)	‎‎22.6±6.2 ‎‎
Family history Positive Negative	2 (3.4) 57 (96.6)
History of surgery Positive Negative	2 (3.4) 57 (96.6)
Smoking status Smoker Non-smoker	3 (5.3) 56 (94.7)
Disease duration (month)	‎‎52.9±56.1 ‎
Phase of disease Flare Remission	‎‎43 (72.9%)‎ (27.1) 16
Disease type UC CD	‎‎‎40 (67.8%) ‎ 19 (32.2%) ‎

IBD: Inflammatory bowel disease; UC: ulcerative colitis; CD: Crohn's disease; Data are shown as mean ± SD or number (%).

## Discussion

Currently, colonoscopy is the mainstay method for the initial diagnosis and assessment of the extent of bowel involvement in IBD (11). Yet, IBD diagnosis still remains a critical challenge for physicians mainly due to their invasiveness as well as their limitations in determining the prognosis, disease activity and severity, and predicting the therapeutic outcomes (3, 12). For this reason, increasing efforts are being made to discover blood-based biomarkers capable of addressing these uncertainties. To date, many blood-based biomarkers have been introduced for IBD (13-16). However, none of them has been proven to be ideal.

In this study, we evaluated the iNOS mRNA expression in the blood leukocytes of IBD patients and compared it with the iNOS mRNA expression in the blood leukocytes of healthy controls to find how this value is different between these groups, as well as how it associates with the disease characteristics in IBD patients. According to our results, the mean iNOS mRNA expression was not significantly different between the IBD patients and healthy controls. However, the mean iNOS mRNA expression was significantly higher in the flare phase of IBD in comparison with the revision phase. 

No significant difference was observed the mean iNOS mRNA expression in the blood leukocytes of CD and UC patients.

Various studies have suggested a pathogenetic role for iNOS and nitric oxide (NO) in IBD. Boughton-Smith *et al*. measured the NO synthase activities in colon mucosa from 11 healthy control subjects, 6 UC patients, and 4 CD patients. Based on their results, NO synthase activity in colonic mucosa of UC patients was about eightfold higher than the value in colonic mucosa of healthy control subjects. However, mucosal NO synthase activity did not differ between the control value and CD patients (17). Lundberg *et al*. directly measured luminal NO in the colons of 12 healthy controls and 6 UC patients in flare. According to their results, the NO concentrations were more than 100 times higher in the UC patients in comparison with the controls subjects (18). Singer *et al. *evaluated the cellular distribution of iNOS in human colonic mucosa from normal bowel, UC, CD, and diverticulitis using immunoperoxidase microscopy with a monospecific human iNOS antibody. The results of this study revealed the intense focal iNOS labeling localized to the inflamed colonic epithelium in UC, CD, and diverticulitis but not in the uninflamed epithelium (19).

With respect to the convincing evidence on the role of iNOS in the pathogenesis of IBD, and considering IBD as a systemic disease with variety of extra-intestinal manifestations (20, 21), it was hypothesized that the increased iNOS level could also be traced in the blood circulation of IBD patients and has the potential to be used as a blood based biomarker. Dijkstra *et al*. studied iNOS expression in the circulating monocytes of 15 patients with active IBD, 6 patients with the remission phase of IBD, and 18 healthy controls using flow cytometry. The results were confirmed by Western blotting and immunocytochemistry. According to their results, the iNOS expression in the circulating monocytes as well as the percentage of iNOS^+^ monocytes was increased in the patients with active IBD in comparison with the healthy controls. This increase was also correlated with the disease activity. Moreover, the patients who went into remission all showed a significant reduction in the iNOS expression (6).

We evaluated the iNOS expression in the blood leukocytes of IBD patients and healthy controls using Real-time PCR, as a more applicable routine laboratory approach. Our results were in accordance with the results of earlier investigations and showed a higher expression of iNOS mRNA in the blood leukocytes of IBD patients in comparison with the healthy controls and in active IBD in comparison with the inactive disease. Yet, we did not find any significant difference between the iNOS mRNA expression of CD and UC patients, while the study of Boughton-Smith *et al*.(17) and Lundberg *et al*.(18) did. 

Our results suggest iNOS as a blood based biomarker for IBD. However, our study has some limitations that should be resolved in future investigations. The main limitation of this study was the small number of patients in the CD and remission group of the patients that could have adversely affected the power of statistical analysis. Thus, future investigations with larger and matched sample size are required to further codify the role of iNOS as a blood-based biomarker of IBD.

## Conflict of interests

The authors declare that they have no conflict of interest.
